# Obesity attenuates D_2_ autoreceptor‐mediated inhibition of putative ventral tegmental area dopaminergic neurons

**DOI:** 10.14814/phy2.12004

**Published:** 2014-05-11

**Authors:** Susumu Koyama, Masayoshi Mori, Syohei Kanamaru, Takuya Sazawa, Ayano Miyazaki, Hiroki Terai, Shinichi Hirose

**Affiliations:** 1 Department of Psychosomatic Medicine, Faculty of Pharmaceutical Sciences, Fukuoka University, Fukuoka, Japan; 2 Department of Pediatrics, School of Medicine, Fukuoka University, Fukuoka, Japan; 3 Central Research Institute for the Pathomechanisms of Epilepsy, Fukuoka University, Fukuoka, Japan

**Keywords:** Brain slices, desensitization, extracellular recording, fat‐rich diets, quinpirole

## Abstract

The ventral tegmental area (VTA) in the midbrain is important for food reward. High‐fat containing palatable foods have reinforcing effects and accelerate obesity. We have previously reported that diet‐induced obesity selectively decreased the spontaneous activity of VTA GABA neurons, but not dopamine neurons. The spontaneous activity of VTA dopamine neurons is regulated by D_2_ autoreceptors. In this study, we hypothesized that obesity would affect the excitability of VTA dopamine neurons via D_2_ autoreceptors. To examine this hypothesis, we compared D_2_ receptor‐mediated responses of VTA dopamine neurons between lean and obese mice. Mice fed on a high‐fat (45%) diet and mice fed on a standard diet were used as obese and lean models, respectively. Brain slice preparations were made from these two groups. Spontaneous activity of VTA neurons was recorded by extracellular recording. Putative VTA dopamine neurons were identified by firing inhibition with a D_2_ receptor agonist quinpirole, and electrophysiological criteria (firing frequency <5 Hz and action potential current duration >1.2 msec). Single‐dose application of quinpirole (3−100 nmol/L) exhibited similar firing inhibition of putative VTA dopamine neurons between lean and obese mice. In stepwise application by increasing quinpirole concentrations of 3, 10, 30, and 100 nmol/L subsequently, quinpirole‐induced inhibition of firing decreased in putative VTA dopamine neurons of obese mice compared with those of lean mice. In conclusion, high‐fat diet‐induced obesity attenuated D_2_ receptor‐mediated inhibition of putative VTA dopamine neurons due to the acceleration of D_2_ receptor desensitization.

## Abstract (precis)

High‐fat diet‐induced obesity did not affect firing properties of putative ventral tegmental area (VTA) dopamine neurons. However, obesity attenuated dopamine D_2_ receptor‐mediated inhibition of putative VTA dopamine neurons due to the acceleration of receptor desensitization. In obesity, decrease in D_2_ receptor‐mediated autoinhibition may contribute to pathophysiology of reinforcing process in feeding.

## Introduction

Recent increases in obesity among children and adolescents are accompanied by a potential risk of metabolic syndrome and type 2 diabetes mellitus. Glycemic impairment in children and adolescents progresses faster compared with adults, in that 25% of severely obese children with impaired glucose tolerance develop type 2 diabetes mellitus within 2 years (Weiss et al. 2005), compared with 5–10 years in adults (Saad et al. 1988). Thus, body weight control by intervention with appropriate diets is critical to prevent obesity, and decrease the future risk for rapidly progressing glycemic impairment in children and adolescents. However, the study by Wheeler et al. (2012) suggests that there is little evidence for a suitable macronutrient composition to manage diabetes mellitus. It is difficult to determine a recommended appropriate diet that obtains an ideal balance among macronutrients such as carbohydrate, protein, and fat. It is easier to determine inappropriate diets, which contain a high fat content and are energy‐rich; causing and accelerating obesity in children and adolescents (Proserpi et al. 1997; McGloin et al. 2002).

Food is a potent natural reward (Volkow et al. 2011), and rodents fail to terminate eating and worsen obesity, and vice versa, when exposed to high‐energy containing diets (la Fleur et al. 2007; Johnson and Kenny 2010). The vicious cycle between excessive consumption of energy‐rich diets and acceleration of obesity is related to the mesolimbic dopamine system in the brain. This reward circuit in the midbrain is mainly composed of the ventral tegmental area (VTA) and nucleus accumbens (NAcb). The excitation of VTA dopamine neurons causes dopamine release in the NAcb, contributing to the reinforcement of natural stimuli (Di Chiara and Imperato 1988; Gonon 1988; Wise 2002; Robinson and Berridge 2003; Fulton 2010; Narayanan et al. 2010). Therefore, consuming salient foods such as high‐fat diets is closely related to the reinforcing process through the central dopaminergic system in obesity. Dopamine activates dopamine receptors, which are mainly divided into D_1_‐like (D_1_ and D_5_) and D_2_‐like receptor subfamilies (D_2_, D_3_, and D_4_), and regulates neuronal excitability through several GTP‐binding protein (G‐protein) coupled ion channels in the brain (De Mei et al. 2009; Luscher and Slesinger 2010). Among these dopamine receptor subfamilies, D_2_ receptors are considered to be important in the pathophysiology of obesity and obesity‐related food reward (Wang et al. 2001; Huang et al. 2005; Johnson and Kenny 2010; Sharma and Fulton 2013). Wang et al. (2001) reported that the number of D_2_ receptors was decreased in the striatum of obese individuals. Johnson and Kenny (2010) reported that palatable food intake decreased D_2_ receptor protein expression in the striatum of obese rats with a change in their feeding behavior. In contrast, Huang et al. (2005) reported that diet‐induced obese mice had significantly higher D_2_ receptor mRNA expression in the NAcb compared with lean and obesity‐resistant mice. Sharma and Fulton (2013) reported that high‐fat diet‐induced obese mice expressed significantly higher D_2_ receptor protein expression in the NAcb compared with lean mice. Although D_2_, D_3_, and D_5_ receptors are present in midbrain dopaminergic neurons (Sesack et al. 1994; Ciliax et al. 2000; Diaz et al. 2000), it remains unclear whether obesity affects neuronal activity through D_2_ receptors in the VTA.

VTA dopamine neurons have intrinsic pacemaker activity, and previous in vivo and in vitro studies have shown that activity of these neurons is regulated by somatodendritic dopamine release (Rice et al. 1997; Kita et al. 2009). In midbrain dopamine neurons, dopamine released from somatodendritic regions activates D_2_ receptors and opens inwardly rectifying K^+^ (IRK) channels via Giα and βγ subunits; outflow of K^+^ ions from the cytoplasm hyperpolarizes membrane potentials and subsequently inhibits neuronal excitability (Kim et al. 1995; Mercuri et al. 1997; Ford et al. 2010; Luscher and Slesinger 2010). We previously reported that spontaneous activity of putative VTA GABA neurons in obese mice decreased compared with lean mice, while activity of putative VTA dopamine neurons was not significantly different between lean and obese mice (Koyama et al. 2013). In this study, we hypothesized that obesity would change the excitability of VTA dopamine neurons through D_2_ autoreceptors. To examine this hypothesis, we used a diet‐induced obese animal model comparable to obese children and adolescents, and identified putative VTA dopamine neurons based on electrophysiological and pharmacological properties. In addition, we compared the D_2_ autoreceptor‐mediated function of these neurons between lean and obese mice using an electrophysiological technique.

## Methods

### Lean and obese mice

Male 4‐week‐old imprinting control region (ICR) mice (Kyudo Co. Ltd., Saga, Japan) were housed in groups (*n* = 5 in a plastic cage; 30 × 25 × 18 cm) with a 12/12‐h light–dark cycle schedule (lights on at 19:00). Mice were kept in a temperature‐ and humidity‐controlled (20–24°C, 53–57%) room under specific pathogen‐free conditions. Mice were given access to food and water ad libitum. Animals used in this study were treated in strict accordance with the U.S. National Institutes of Health Guide for the Care and Use of Laboratory Animals, and all experimental methods were approved by the Animal Care Committee of Fukuoka University.

One group of mice aged 4 weeks was fed a standard diet (fat 13%, carbohydrate 60%, and protein 27%, total energy 3.5 kcal/g) (CE‐2: Clea Japan Inc., Tokyo, Japan) for 5–6 weeks to obtain lean models. The other group of mice was fed a high‐fat diet (fat 45%, carbohydrate 35%, and protein 20%, total energy 4.7 kcal/g) (D12451; Research Diets, New Brunswick, NJ) for the same period to obtain obese models. At 9–10 weeks of age, obese mice exhibit higher body weight with greater accumulation of visceral adipose tissues compared with lean mice (Koyama et al. 2013).

### Preparation of brain slices

Under anesthesia with pentobarbital (50 mg/kg), each mouse (9–10 weeks old) was killed and the brain quickly removed. The brain was placed in an ice‐cold cutting solution consisting of (in mmol/L): 220 sucrose, 2.5 KCl, 2.4 CaCl_2_, 1.3 MgSO_4_, 1.24 NaH_2_PO_4_, 26 NaHCO_3_, 11 D‐glucose, and 0.4 ascorbic acid, which was constantly bubbled with 95% O_2_ and 5% CO_2_. A transverse brain slice, at a thickness of 400 μm, was cut using a vibrating blade brain slicer (7000 SMZ, Campden Instruments, Loughborough, UK). Brain slices were incubated in an artificial cerebrospinal fluid (ACSF), consisting of (mmol/L): 126 NaCl, 2.5 KCl, 2.4 CaCl_2_, 1.3 MgSO_4_, 1.24 NaH_2_PO_4_, 26 NaHCO_3_, and 11 D‐glucose, which was constantly bubbled with 95% O_2_ and 5% CO_2_ at room temperature (20‐25°C) for at least 1 h. Then, the brain slice was placed on a glass platform in a recording chamber (RC‐22C, Warner Instruments, Hamden, CT) and perfused with the ACSF, which was constantly bubbled with 95% O_2_ and 5% CO_2_. The ACSF was warmed to 35°C using an in‐line solution heater, which was connected to a thermostatic temperature circulator (NTT‐2200, Tokyo Rikakikai Co. Ltd., Tokyo, Japan). The temperature of the ACSF in the recording chamber was directly monitored using a digital thermometer (7001H, Netsuken, Tokyo, Japan). An S‐shaped platinum frame was used to hold the brain slice in the recording chamber. The VTA, located between the interfascicular nucleus and the medial lemniscus in the horizontal axis, and between the paranigral nucleus and the red nucleus in the sagittal axis, was visually identified (Franklin and Paxinos 2008) under a binocular dissection microscope (M50, Leica, Solms, Germany).

### Electrophysiological recording

After approximately 1 h of perfusion with ACSF, blind extracellular recordings of the brain slice preparation were performed. Extracellular voltage‐clamp recording at a holding potential of 0 mV is able to avoid disrupting the intracellular milieu, and provides a low impedance pathway through the patch for fast events occurring in a few ms, such as action potentials (APs) (Perkins 2006). Spontaneous AP currents were recorded using a Multiclamp‐700B patch‐clamp amplifier (Molecular Devices, Sunnyvale, CA). Microelectrodes were fabricated from glass capillaries (O.D.: 1.5 mm, I.D.: 0.86 mm) (BF150‐86‐10, Sutter Instrument Company, Novato, CA) on a P‐97 puller (Sutter Instrument Company). The tip resistance of each electrode was 3–9 MΩ when filled with 0.9% NaCl. A depolarizing rectangular voltage pulse of 10 mV was applied to the electrode and negative pressure was gently applied to complete a loose patch. Seal resistance was 40–200 MΩ and was periodically monitored during recordings. Membrane currents were filtered at 2 kHz and acquired at a sampling frequency of 10 kHz. Data acquisition was performed using a Digidata 1440A interface and pClamp software version 10.2 (Molecular Devices).

### Identification of putative VTA dopamine neurons

Putative VTA dopamine neurons were identified in accordance with electrophysiological and pharmacological properties. These neurons had AP current duration >1.2 ms and firing frequency (FF) <5 Hz (Koyama et al. 2013) and were inhibited by the application of the D_2_ receptor agonist, quinpirole (Sigma‐Aldrich, St. Louis, MO). VTA neurons, which simultaneously met these three criteria, were used for analyses in this study.

### Quinpirole application

Brain slices were continuously perfused with ACSF and quinpirole were dissolved at final concentrations in the same solution. Quinpirole solutions were applied by a multiple channel manifold (MP‐5, Warner Instruments). Each channel of the manifold was connected to a gravity‐fed reservoir with tubing. Solution flowed constantly through one manifold channel connected to the recording chamber. Application of quinpirole solutions was controlled by opening or closing valves connected to the reservoirs. In single‐dose quinpirole application, interval between the applications was set to be 20 min to prevent D_2_ receptor desensitization. In stepwise quinpirole application, 10, 30, and 100 nmol/L quinpirole was applied subsequently.

### Data analysis and statistics

AP currents were detected by their peaks of initial inward current component using a threshold‐searching configuration in pClamp software (Molecular Devices). Duration between the peaks was estimated to be the interspike interval (ISI), and FF was also calculated. The coefficient of variation (CV) of the ISI was obtained by dividing the standard deviation of the ISIs by the mean ISI. AP current duration was measured between the initiation of the inward current component and subsequent outward current peak of the AP current, as previously described (Koyama et al. 2013). Records including an AP current amplitude <10 pA and CV of ISI > 1.0 were considered unstable and not used for analyses. Records were also excluded from analyses when spontaneous firing did not recover after washout of quinpirole. Each concentration of quinpirole was applied for four min. In application of quinpirole, each 1‐min‐long FF epoch was normalized to the average four epochs just before drug application. Concentration‐response curves for quinpirole‐induced inhibition in lean and obese mice were constructed by plotting normalized FF as a function of drug concentration plotted on a logarithmic scale. The normal distribution of the data was evaluated using the Kolmogorov‐Smirnov test. When normality was confirmed, two‐tailed Student's t‐tests were used for comparisons between the two groups. Significant differences in quinpirole‐induced responses between lean and obese mice were determined using two‐way ANOVA followed by a Bonferroni correction. Differences were considered statistically significant at *P *<**0.05. Numerical values are reported as mean ± standard error of the mean (SEM). Graphing and statistical analyses were conducted using Origin8 software (OriginLab, Northampton, MA).

## Results

### Firing inhibition by single‐dose quinpirole application

**Figure 1.**
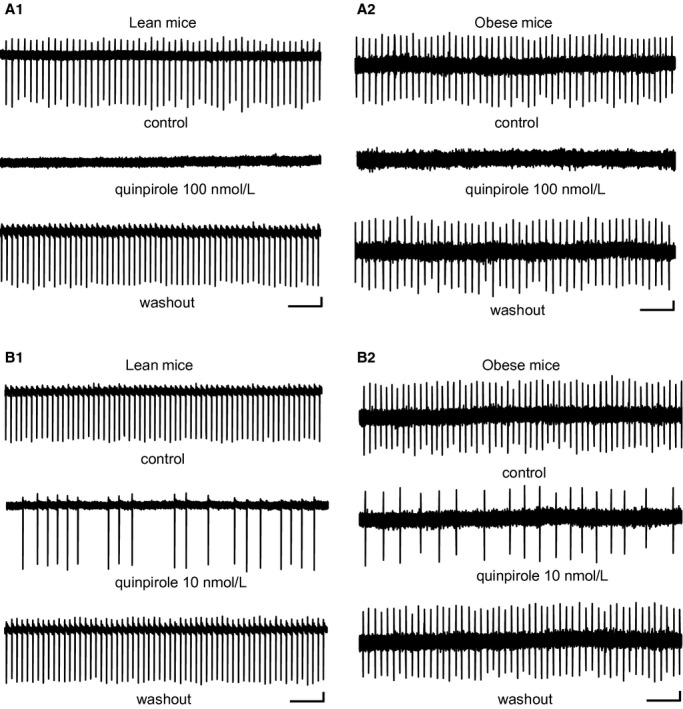
Single‐dose application of quinpirole to VTA neurons in lean and obese mice. (A1) Spontaneous firing records before, during, and after application of 100 nmol/L quinpirole in lean mice. Scale bars, 20 pA; 2 sec. (A2) Spontaneous firing records before, during, and after application of 100 nmol/L quinpirole in obese mice. Scale bars, 10 pA; 2 sec. (B1) Spontaneous firing records before, during, and after application of 10 nmol/L quinpirole in lean mice. Scale bars, 20 pA; 2 sec. (B2) Spontaneous firing records before, during, and after application of 10 nmol/L quinpirole in obese mice. Scale bars, 10 pA; 2 sec.

**Figure 2.**
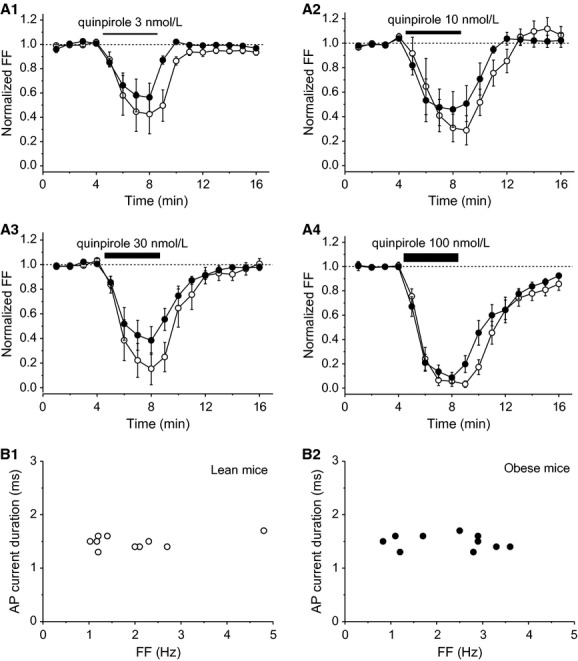
Quinpirole‐induced inhibition of putative VTA dopamine neurons in lean and obese mice. Time courses of normalized FF of VTA neurons in lean (open circles) and obese mice (closed circles) before, during, and after single‐dose application of quinpirole: 3 nmol/L (A1) (lean mice, *n* = 6; obese mice, *n* = 6), 10 nmol/L, (A2) (lean mice, *n* = 9; obese mice, *n* = 7), 30 nmol/L (A_3_) (lean mice, *n* = 6; obese mice, *n* = 9), 100 nmol/L (A4) (lean mice, *n* = 10; obese mice, *n* = 10). Each point represents FF for 1 min. Relationship between FF and AP current duration of the VTA neurons in lean (C1) (open circles, *n* = 10) and obese mice (C2) (closed circles, *n* = 10). Values are mean ± SEM.

In this study, obese mice had significantly higher body weight (47.3 g ± 1.3, *n* = 9) compared with lean mice (40.7 g ± 0.6, *n* = 14) (*P *<**0.0001). Figure 1 shows spontaneous firing of VTA neurons when single‐dose quinpirole application was conducted. In both lean and obese mice, 100 nmol/L quinpirole blocked spontaneous firing (Fig. 1A1 and A2) and 10 nmol/L quinpirole reduced FF in a similar extent (Fig. 1B1 and B2). Figure 2 shows average time courses of FF of VTA neurons before, during, and washout of quinpirole in single‐dose application. In lean and obese mice, quinpirole inhibited FF of VTA neurons in a concentration‐dependent manner (Fig. 2A1–A4). The peak FF inhibition rate with quinpirole was similar between lean and obese mice. It is noted that recovery time of FF after washout of quinpirole was shorter in VTA neurons of obese mice compared with those of lean mice. The VTA neurons examined by the single‐dose application protocol met the criteria for putative dopamine neurons in lean (Fig. 2B1) and obese mice (Fig. 2B2). FF of putative VTA dopamine neurons was 2.0 ± 0.4 Hz in lean mice (*n* = 10) and 2.3 ± 0.3 Hz in obese mice (*n* = 10); there was no significant difference between the two groups. AP current duration of putative VTA dopamine neurons was 1.49 ± 0.04 ms in lean mice (*n* = 10) and 1.49 ± 0.04 ms in obese mice (*n* = 10); there was no significant difference between the two groups.

### Firing inhibition by stepwise quinpirole application

**Figure 3.**
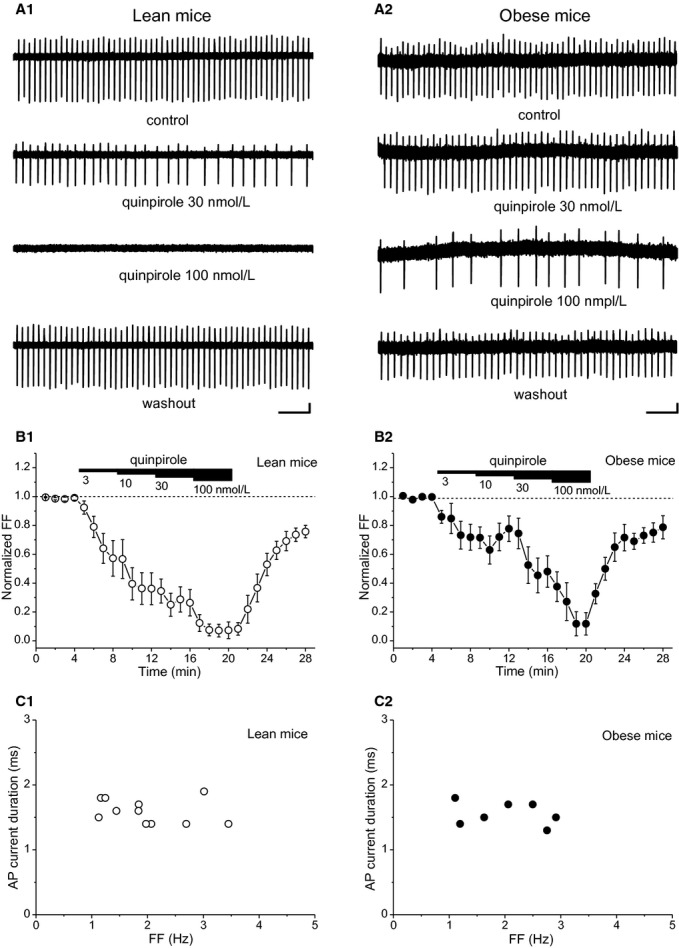
Stepwise application of quinpirole to putative VTA dopamine neurons in lean and obese mice. Spontaneous firing of VTA neurons before, during, and, after application of quinpirole (30 and 100 nmol/L) in lean mice (A1) and obese mice (A2). Time courses of normalized FF before, during, and after stepwise application of quinpirole in lean mice (open circles, *n* = 11) (B1) and obese mice (closed circles, *n* = 7) (B2). Relationship between FF and AP current duration of the VTA neurons in lean mice (open circles, *n* = 11) (C1) and obese mice (closed circles, *n* = 7) (C2). Values are mean ± SEM.

We next examined the spontaneous firing of VTA neurons using quinpirole by stepwise application increasing quinpirole concentrations of 3, 10, 30, and 100 nmol/L (Fig. 3). In lean mice, quinpirole with concentration of 100 nmol/L ceased spontaneous firing (Fig. 3A1). In contrast, the same concentration of quinpirole did not cease spontaneous firing in obese mice (Fig. 3A2). Figure 2B shows the time courses of quinpirole‐induced inhibition in VTA neurons from lean and obese mice. In obese mice, desensitization of quinpirole‐induced inhibition was prominent by subsequently applied quinpirole. Quinpirole‐induced inhibition of FF was significantly decreased in obese mice (Fig. 3B2) compared with lean mice (Fig. 3B1). (*P *<**0.0001, two‐way ANOVA) The VTA neurons examined by the stepwise application protocol met the criteria for putative dopamine neurons in lean (Fig. 3C1) and obese mice (Fig. 3C2). FF of putative VTA dopamine neurons was 2.0 ± 0.2 Hz in lean mice (*n* = 11) and 2.0 ± 0.3 Hz in obese mice (*n* = 7); there was no significant difference between the two groups. AP current duration of putative VTA dopamine neurons was 1.60 ± 0.07 ms in lean mice (*n* = 11) and 1.57 ± 0.07 ms in obese mice (*n* = 7); there was no significant difference between the two groups.

### Quinpirole‐induced firing inhibition by single‐dose and stepwise application

**Figure 4.**
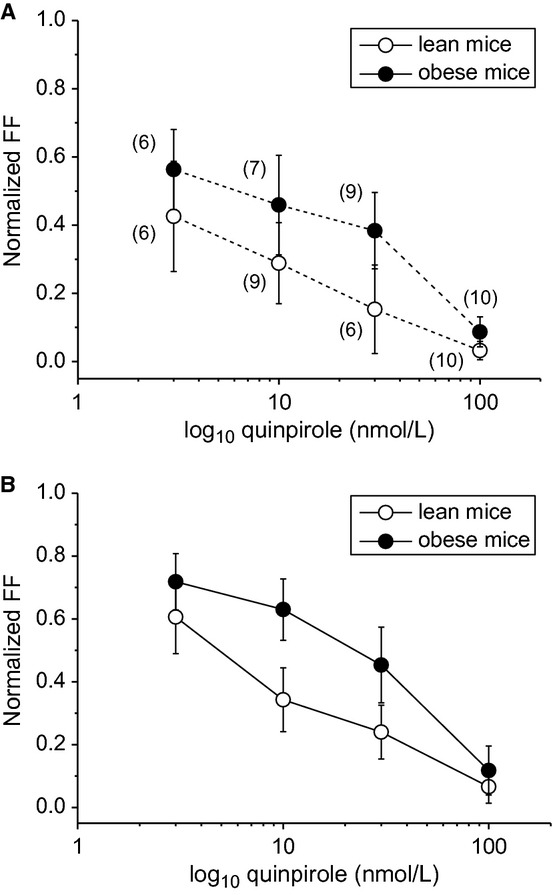
Relationship between quinpirole concentrations and normalized FF in lean and obese mice. (A) Single‐dose quinpirole application protocol for lean (open circles) and obese mice (closed circles). The number of neurons is presented in parentheses. (B) Stepwise quinpirole application protocol for lean (open circles, *n* = 11) and obese mice(closed circles, *n* = 7). *X*‐axes are presented on a logarithmic scale. Values are mean ± SEM.

As shown in Fig. 4, we examined the relationship between quinpirole concentration and FF inhibition of putative VTA dopamine neurons from lean and obese mice in single‐dose application and stepwise application protocols. In single‐dose quinpirole application, there was no significant difference between quinpirole‐induced FF inhibition of putative VTA dopamine neurons between lean and obese mice (*P > *0.05) (Fig. 4A). In stepwise quinpirole application, quinpirole‐induced FF inhibition significantly decreased in putative VTA dopamine neurons of obese mice compared with those of lean mice (*P *<**0.01) at quinpirole concentrations of 10 and 30 nmol/L (Fig. 4B).

## Discussion

In this study, we found that D_2_ receptor‐mediated inhibition of putative VTA dopamine neurons was significantly decreased in obese mice compared with lean mice using an electrophysiological technique.

One of the conventional properties of putative VTA dopamine neurons is firing inhibition by D_2_ autoreceptor activation (Johnson and North 1992; Mercuri et al. 1997). However, this pharmacological property is not always reliable for the identification of putative dopamine neurons in the VTA. Previous studies, using retrograde tracers, have shown that whether dopamine receptor agonists inhibit the firing of VTA dopamine neurons through D_2_ receptors is related to the difference in their dopaminergic projections in the brain (Lammel et al. 2008; Margolis et al. 2008). Lammel et al. (2008) reported that VTA dopamine neurons projecting to the prefrontal cortex did not possess somatodendritic D_2_ autoreceptors. In addition, Margolis et al. (2008) reported that VTA dopamine neurons projecting to the amygdaloid complex were not inhibited by quinpirole. These previous studies show that VTA dopamine neurons, which selectively provide afferents to the NAcb, have somatodendritic D_2_ autoreceptors and are inhibited by dopamine receptor agonists (Lammel et al. 2008; Margolis et al. 2008). The electrophysiological characteristics of VTA neurons, slow FF with a broad AP duration, are not always reliable indicators of putative dopamine neurons (Cohen et al., 2012; Lammel et al. 2008; Margolis et al. 2008). Our previous study provided additional electrophysiological criteria, which more reliably identified putative VTA dopamine neurons (Koyama et al. 2013), while supporting the previous criteria (Ungless et al. 2004; Ford et al. 2006; Chieng et al. 2011; Luo et al. 2011). In our criteria, putative VTA dopamine neurons of lean and obese mice had a FF < 5 Hz and AP current duration >1.2 msec (Koyama et al. 2013). In this study, VTA neurons of lean and obese mice met these electrophysiological criteria, in addition to being inhibited with dopamine receptor agonists. Therefore, VTA neurons of lean and obese mice in this study are considered to be putative dopamine neurons, and may correspond to the mesoaccumbens projection neurons in the VTA.

In our previous study, the FF of putative VTA GABA neurons in obese mice was decreased significantly compared with lean mice, suggesting that diet‐induced obesity decreases the excitability of these neurons by directly modulating ion channel properties (Koyama et al. 2013). In this study, firing properties of putative VTA dopamine neurons were not significantly different between lean and obese mice, suggesting that high‐fat diet‐induced obesity does not affect ion channel properties, which contribute to the excitability of these neurons.

In this study, quinpirole inhibited the FF of putative VTA dopamine neurons in a concentration‐dependent manner in lean and obese mice. Although quinpirole is a D_2_/D_3_ receptor agonist, selective D_2_ receptor activation is likely to inhibit the FF of putative VTA dopamine neurons in lean and obese mice. Davila et al. (2003) reported that D_3_ receptors of midbrain dopaminergic neurons did not activate G‐protein‐coupled IRK channels, suggesting that D_3_ receptor activation with quinpirole fails to inhibit the FF of VTA dopamine neurons. In this study, using single‐dose and stepwise quinpirole application protocols, we found that receptor desensitization rather than receptor hyposensitivity to the agonist contribute to the attenuation of D_2_ autoreceptor function in putative VTA dopamine neurons in obese mice. Since Sharma and Fulton (2013) reported that diet‐induced obesity did not affect D_2_ receptor protein expression in the VTA, it is likely that changes in intracellular signal transduction from D_2_ autoreceptors to IRK channels via G‐protein are involved in the acceleration of D_2_ receptor desensitization in obesity. Previous studies have reported cellular mechanisms on D_2_ receptor desensitaization (Namkung and Sibley 2004; Bartlett et al. 2005). Bartlett et al. (2005) reported that G‐protein coupled receptor‐associated sorting protein contributed to D_2_ receptor desensitization in rat brain slice preparation. Namkung and Sibley (2004) reported that one of the two intracellular domains (domain I and II) of a D_2_ autoreceptor contributed to receptor desensitization when the domain I was phosphorylated with protein kinease C.

In this study, diet‐induced obesity did not affect spontaneous activity of putative VTA dopamine neurons, while diet‐induced obesity attenuated D_2_ receptor‐mediated inhibition of putative VTA dopamine neurons. The potency of D_2_ receptor function was attenuated with receptor desensitization at 10‐30 nmol/L quinpirole within 20 min. The changes found in putative VTA dopamine neurons from high‐fat diet mice in this study would be expected to increase dopamine transmission to target regions, especially in the NAcb, as decrease in function of D_2_ autoreceptors. In obesity, VTA dopamine neurons would compensate for the decreased D_2_ receptor‐mediated inhibition by increasing somatodendritic dopamine release, while dopamine synthesis in VTA dopamine neurons would be shrinking (Li et al. 2009), decreasing dopamine transmission from nerve terminals in the NAcb (Geiger et al. 2009) over the long‐term excessive dopamine synthesis and release in these neurons. This scenario is supported by the results that the previous studies exposed animals to high‐fat diet for about 14 weeks, while we exposed mice to high‐fat diet for 5‐6 weeks. However, it is noted that this explanation on the difference in dopaminergic function between the VTA and the NAcb in obesity should be examined by further studies. In conclusion, we have shown that high‐fat diet‐induced obesity attenuated D_2_‐receptor‐mediated inhibition of putative VTA dopamine neurons due to D_2_ receptor desensitization. The decrease in D_2_ autoreceptor function of VTA dopamine neurons may contribute to the pathophysiology of reward‐related feeding behavior in obesity.

## Acknowledgments

The authors thank Dr. Mark S. Brodie, Department of Physiology and Biophysics, University of Illinois, Chicago for his helpful comments on this manuscript.

## Conflict of interest

None declared.
